# Feasibility of the Drill Dilator as an Initial Dilation Device for Postoperative Biliary Strictures with Surgically Altered Anatomy

**DOI:** 10.3390/jcm15218666

**Published:** 2026-11-06

**Authors:** Kenta Yoshida, Satoshi Sato, Chikara Iino, Kota Fujimaki, Keita Mikami, Keisuke Furusawa, Satoru Tsuruta, Yusuke Wakasa, Hayato Nagase, Norihisa Kimura, Keinosuke Ishido, Hirotake Sakuraba

**Affiliations:** 1 Department of Gastroenterology, Hematology, and Clinical Immunology, Hirosaki University Graduate School of Medicine, Hirosaki 036-8562, Japan; kyoshida@hirosaki-u.ac.jp (K.Y.);; 2 Department of Gastroenterological Surgery, Hirosaki University Graduate School of Medicine, Hirosaki 036-8562, Japan

**Keywords:** drill dilator, postoperative biliary stricture, surgically altered anatomy, endoscopic treatment

## Abstract

**Background:** Endoscopic treatment of postoperative biliary strictures in patients with surgically altered anatomy can be technically challenging. The Drill Dilator advances by rotational torque with minimal forward pushing, potentially facilitating dilation while maintaining endoscope stability. This study evaluated the feasibility and safety of the Drill Dilator as an initial dilation device for postoperative biliary strictures in patients with surgically altered anatomy. **Methods:** We retrospectively reviewed 46 endoscopic sessions for postoperative biliary strictures performed between December 2016 and April 2026. Among these, 18 initial treatment sessions were included in the comparative analysis. Six sessions in which the Drill Dilator was used as the initial dilation device were evaluated and compared with 12 sessions using conventional dilation devices. **Results:** The Drill Dilator successfully passed and dilated the stricture in all sessions (100%). Additional dilation with another device was required in 50% of sessions (3/6). Technical success was achieved in all sessions (100%). Mild post-ERCP cholangitis (PEC) occurred in two patients (33.3%), with no severe procedure-related adverse events. In the conventional dilation group, device passage and technical success rates were 100% (12/12) and 91.7% (11/12), respectively. **Conclusions:** The Drill Dilator was feasible as an initial dilation device for postoperative biliary strictures in patients with surgically altered anatomy. Its use may facilitate subsequent endoscopic dilation, particularly in situations where alignment between the dilation device and the bile duct is challenging.

## 1. Introduction

Surgical treatment for hepatopancreatobiliary disease frequently requires biliary and/or intestinal reconstruction, and postoperative complications such as anastomotic stricture and bile leakage are important clinical issues. Recent advances in balloon enteroscopy and endoscopic devices have enabled endoscopic intervention to become the primary treatment for postoperative biliary strictures in patients with surgically altered anatomy. However, endoscopic intervention for postoperative biliary strictures is often technically challenging because of the technical difficulty of endoscopic manipulation, anatomical location of the hepaticojejunostomy, and steep angle of the bile duct. A treatment strategy, including appropriate device selection, has not been established. A recent systematic review and meta-analysis reported overall endoscopy success rate of 89% for double-balloon enteroscopy-assisted ERCP in patients with surgically altered anatomy. However, successful access to the biliary anastomosis does not necessarily ensure successful treatment of severe biliary strictures [1].

Previous studies have demonstrated the feasibility of balloon dilation combined with plastic stent placement for hepaticojejunostomy anastomotic strictures. In a prospective multicenter study, Tomoda et al. reported a technical success rate of 97.5% using this approach [2]. Conventional dilators (balloon/bougie) have traditionally been used for endoscopic dilation of postoperative biliary strictures in patients with surgically altered anatomy. However, these devices primarily rely on forward pushing force for advancement, and the resulting reactive force from the stricture may destabilize the endoscope during dilation.

The Tornus ES (Asahi Intec, Aichi, Japan), known as a Drill Dilator, is a novel endoscopic dilation device. Although its usefulness has been reported for tract dilation during interventional endoscopic ultrasound (EUS) procedures [3], usefulness for postoperative biliary strictures is limited to a few case reports. Because the Drill Dilator may facilitate dilation with minimal forward pushing force, it may be particularly advantageous in patients with surgically altered anatomy. However, the feasibility of selecting the Drill Dilator as the initial dilation device, rather than using it as a rescue device after failure of dilation by conventional dilator, has not been sufficiently investigated.

This study aimed to evaluate the feasibility, safety, and clinical characteristics of the Drill Dilator as an initial dilation device for postoperative biliary strictures in patients with surgically altered anatomy.

## 2. Methods

### 2.1. Patients (Figure 1)

**Figure 1:**
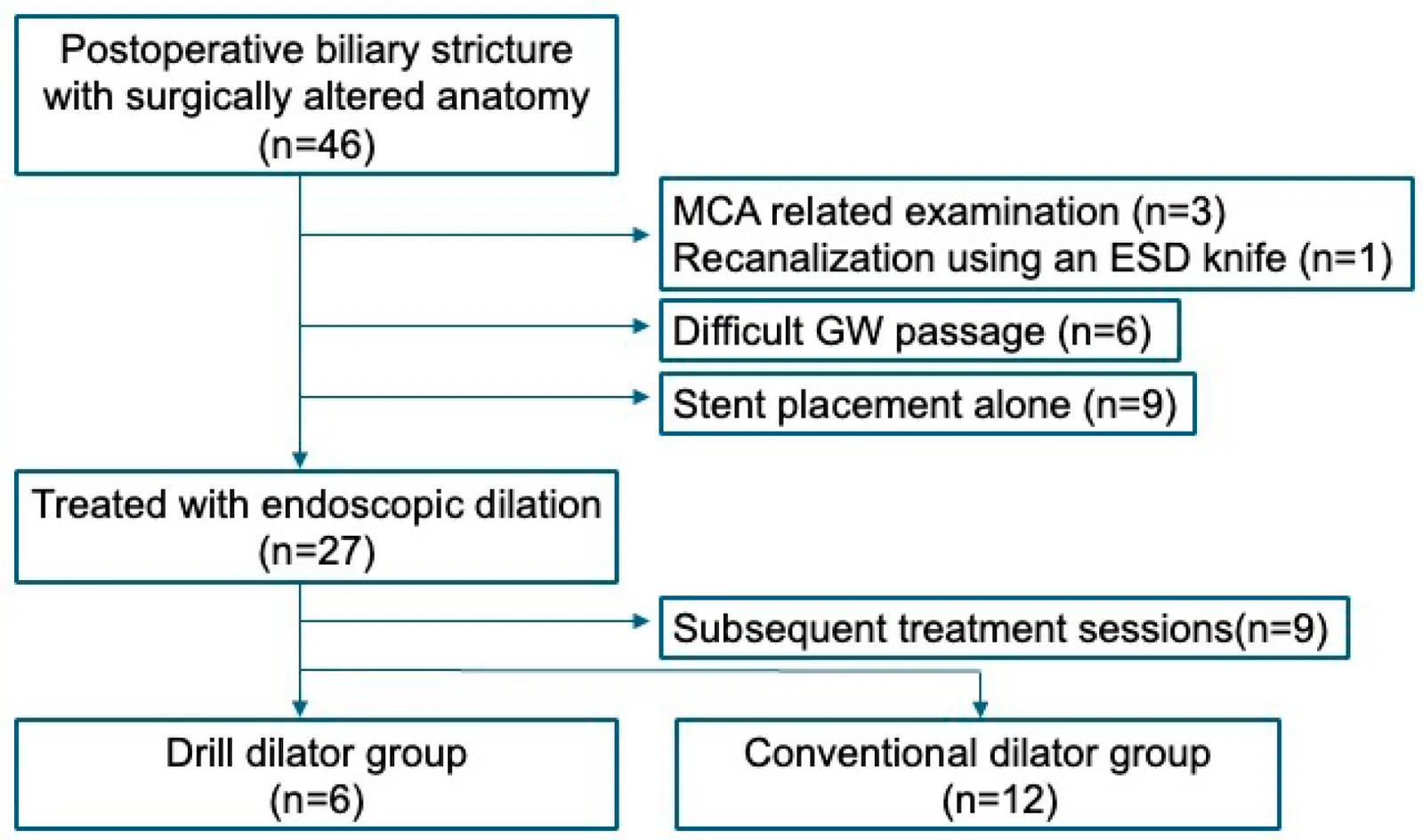
Flowchart of patient selection.

This was a single-center retrospective study. Between December 2016 and April 2026, a total of 46 endoscopic sessions for postoperative biliary strictures in patients with surgically altered anatomy were performed at our institution. Sessions involving magnetic compression anastomosis (MCA) and related procedures, ESD knife recanalization for complete obstruction, unsuccessful guidewire passage, or stent placement alone were excluded. A total of 27 sessions involving endoscopic dilation were identified. Of these, only the initial treatment session for each patient was included in the study. The included sessions were divided into the Drill Dilator group (*n* = 6) and the conventional dilator group (*n* = 12) according to the initial dilation device used. Initial use was defined as the use of a dilation device as the first device for stricture dilation after successful guidewire passage across the stricture, without prior attempts at dilation using another device Figure 1.

**Endpoints**

Primary endpoint

Dilation device passage

Secondary endpoints

Technical success

Need for additional dilation

Procedure time

Adverse events

Recurrence of strictures

Dilation device passage was defined as successful advancement of the widest portion of the device across the stricture for the Drill Dilator and bougie dilators, or adequate expansion of the central portion of the balloon at the stricture site for balloon dilators.

Technical success was defined as successful completion of the planned endoscopic treatment including stent placement when clinically indicated. Adverse events were classified according to the American Society for Gastrointestinal Endoscopy (ASGE) lexicon [4].

### 2.2. Procedure

(1) Tornus ES (Video S1)

The Tornus ES (Asahi Intec, Aichi, Japan) is a Drill Dilator for endoscopic procedures that was approved for reimbursement in Japan in 2022. It is available in two models compatible with 0.025-inch and 0.018-inch guidewires, both of which provide dilation up to approximately 7 Fr. The Tornus ES advances by rotational torque rather than forward pushing. During the procedure, an assistant rotates the proximal handle clockwise, allowing the device to pass and dilate the stricture without requiring excessive forward force by the endoscopist. After dilation, the device can be smoothly withdrawn by rotating the proximal handle counterclockwise.

**Figure 2:**
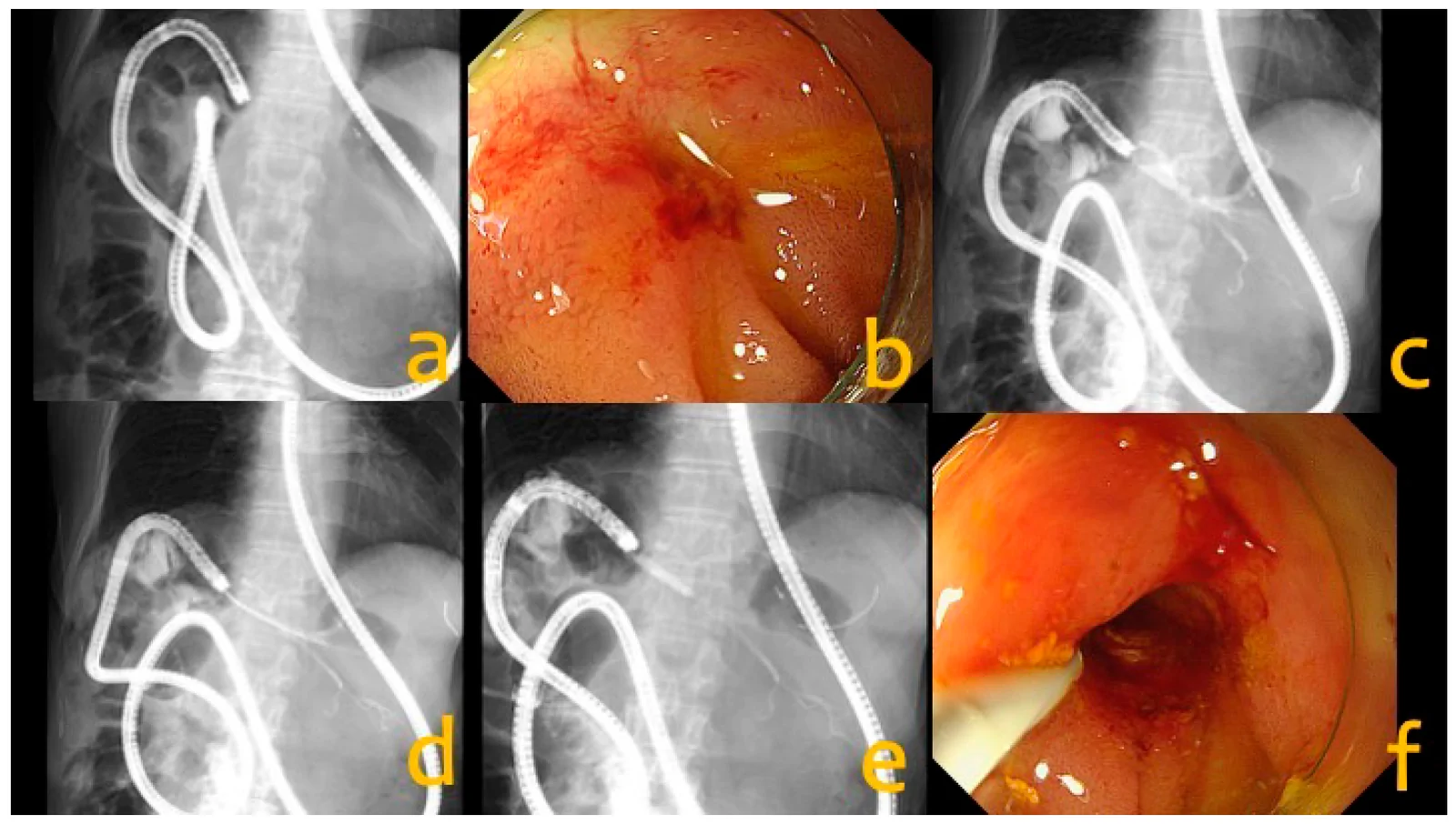
Endoscopic dilation to biliary stricture with surgically altered anatomy. (**a**) Endoscopic evaluation using a single-balloon enteroscope; (**b**) A severe hepaticojejunostomy anastomotic stricture was identified; (**c**) A guidewire was successfully advanced across the stricture; (**d**) Stricture passage and dilation were performed using the Drill Dilator; (**e**) Additional dilation was performed using a balloon dilator; (**f**) Satisfactory dilation of the stricture was achieved.

(2) Endoscopic approach to postoperative biliary strictures with surgically altered anatomy (Figure 2)
  ① Patients with a history of surgery for hepatopancreatobiliary disease who were suspected of having a postoperative biliary stricture underwent endoscopic evaluation. Patients with recurrent cholangitis or peripheral bile duct dilation on computed tomography (CT) were frequently evaluated for suspected postoperative biliary strictures. In patients with a long surgically reconstructed intestinal limb, balloon enteroscopy was used to assess the biliary anastomosis (Figure 2a).
  ② After the scope reached the hepaticojejunostomy or the duodenal papilla, the anastomosis and bile duct were carefully evaluated for the presence of a stricture. When a stricture was identified, gentle guidewire manipulation was performed to achieve guidewire passage across the stricture (Figure 2b). If guidewire passage was unsuccessful because of severe stenosis, alternative approaches, including interventional EUS, percutaneous transhepatic biliary drainage (PTBD), or magnetic compression anastomosis (MCA) [5], were considered.
  ③ If the guidewire successfully crossed the stricture (Figure 2c), endoscopic dilation was performed. In cases cholangiography catheter could not be advanced because of severe stenosis, a dilator (Drill/Balloon/Bougie) was used (Figure 2d). The choice of the initial dilation device was at the discretion of the endoscopist, and no predefined criteria were used for device selection. In all six patients in the Drill Dilator group, the Drill Dilator was selected as the first dilation device without prior attempted using conventional devices. If adequate dilation was not achieved under fluoroscopic guidance using a single dilation device, additional dilation using another device was performed (Figure 2e).
  ④ After confirming adequate dilation of the stricture (Figure 2f), stent placement was not routinely performed during the initial intervention. However, in cases with technically challenging strictures or recurrent strictures, a plastic stent was additionally placed to facilitate future endoscopic reintervention. In these cases, a 5-Fr or 7-Fr plastic stent of sufficient length to bridge the stricture was placed.

## 3. Results

The Patient Background of the Drill Dilator group and the conventional dilator group are summarized in Table 1. The most common primary disease was bile duct cancer, and the most common surgical procedure was subtotal stomach-preserving pancreaticoduodenectomy (SSPPD) with II-A-1 reconstruction in both groups.

The procedural outcomes of the Drill Dilator or the conventional dilator groups are summarized in Table 2. The stricture sites (hepaticojejunostomy/distal bile duct) were distributed as follows: 6/0 in the Drill Dilator group and 11/1 in the conventional dilator group. The etiologies of the strictures (benign/malignant) were distributed as follows: 5/1 in the Drill Dilator group and 11/1 in the conventional dilator group. In the conventional dilator group, balloon dilators were used in eight patients and bougie dilators in four patients. Guidewire passage across the stricture was successfully achieved in all sessions. Following successful guidewire passage, successful passage of the dilation device was achieved in all cases with both the Drill Dilator and the conventional dilator. Technical success was not achieved in one patient in the conventional dilator group. In this patient, both bougie and balloon dilators successfully traversed the stricture; however, placement of even a small-caliber 5-Fr plastic stent was unsuccessful. The Drill Dilator was not used as a rescue device in this patient. Additional dilation was required in 50.0% (3/6) of patients in the Drill Dilator group, although technical success was achieved in 100% (6/6). In all three patients requiring additional dilation in the Drill Dilator group, a balloon dilator was used as the second dilation device.

The median total procedure times were 81 min (range, 33–125 min) and 79 min (range, 20–174 min) in the Drill Dilator and conventional dilator groups, respectively. The median dilation procedure times were 62 min (range, 28–98 min) and 46 min (range, 10–127 min) respectively.

The two cases of mild post-ERCP cholangitis (PEC) in the Drill Dilator group resolved promptly with antibiotic treatment. In the conventional dilator group, procedure-related adverse events occurred in two patients (16.7%): one case of mild PEC and one case of moderate post-ERCP pancreatitis. No severe adverse events were observed in either group. The median follow-up periods were 247.5 days (range, 18–589 days) in the Drill Dilator group and 920.5 days (range, 2–3344 days) in the conventional dilator group. No restenosis requiring repeat dilation was observed, except in one patient with malignant local recurrence in the conventional dilator group.

## 4. Discussion

The Drill Dilator is an endoscopic dilation device used for tract dilation during interventional EUS and for the treatment of postoperative biliary strictures. Because of its potential ability to traverse severe strictures, it is generally used as a rescue device for severe strictures that cannot be passed with conventional dilation devices. Recently, its properties have been applied in various challenging situations. Almasri et al. reported successful Y-configuration biliary stent placement by dilating the mesh of a previously placed self-expandable metal stent (SEMS) [6]. Kawaji et al. reported successful antegrade SEMS placement after dilation of a distal biliary stricture via an EUS-guided hepaticogastrostomy route [7].

The Drill Dilator advances by rotational torque rather than axial pushing forces. It minimizes backward movement of the endoscope during dilation and helps maintain a stable endoscopic position [3]. This characteristic is particularly advantageous during interventional EUS procedures because stable scope positioning is often difficult to maintain. Ogura et al. reported that compared with a conventional bougie dilator, the Drill Dilator achieved a higher technical success rate, and a shorter procedure time during EUS-guided pancreaticogastrostomy [8]. Sadek et al. reported the safety and efficacy of the Drill Dilator for tract dilation during EUS-guided pancreaticojejunonostomy [9].

Although the usefulness of the conventional dilation devices for postoperative biliary strictures in patients with surgically altered anatomy has been reported [10,11], evidence regarding the use of the Drill Dilator remains limited to a few case reports. Toyonaga et al. reported successful dilation of severe biliary strictures using the Drill Dilator after conventional devices failed to pass the strictures [12]. Okamoto et al. reported successful dilation of a hepaticojejunostomy anastomotic stricture using the Drill Dilator during balloon enteroscopy-assisted ERCP [13]. In contrast to the rescue use described in previous report, this study evaluated the Drill Dilator as the initial dilation device after successful guidewire passage, without prior attempts at dilation using conventional devices. Endoscopic treatment in patients with surgically altered anatomy is often technically challenging, because advancement of the endoscope through the reconstructed tract can be difficult, and the axis of the dilation device may not coincide with that of the bile duct [14]. Once guidewire passage had been achieved, the Drill Dilator successfully passed and dilated the stricture in all sessions (100%, 6/6), and technical success was also achieved in all sessions (100%, 6/6). No severe adverse events occurred; however, mild post-ERCP cholangitis (PEC) was observed in two of six patients (33.3%). The direct causal relationship between these events and dilation using the Drill Dilator could not be determined in this study. The Drill Dilator advances by rotational torque. The rotational mechanism of the Drill Dilator may facilitate dilation in situations where alignment between the dilation device and the bile duct is difficult. However, endoscope stability was not objectively assessed in this study, and its potential advantage in maintaining endoscope stability remains hypothetical.

Previous studies have described balloon dilation as a first-line treatment for hepaticojejunostomy anastomotic strictures, with plastic stent placement considered for refractory cases [15]. This study focused on the selection of the initial dilation device after successful guidewire passage. The Tornus ES has a maximum shaft diameter equivalent to 7 Fr and is designed to dilate strictures up to this caliber. However, subsequent stent placement may occasionally remain difficult despite successful passage. Because of its screw-shaped design, the Drill Dilator has a relatively small contact area with the stricture, and the grooves between the threads may reduce the radial dilation force [16]. Therefore, if adequate dilation cannot be achieved with the Drill Dilator alone, additional dilation using another device may facilitate completion of the procedure. Nevertheless, once the Drill Dilator has successfully crossed the stricture, it establishes an initial tract through which subsequent dilation can be performed. The Drill Dilator should not be regarded as a replacement for conventional dilation devices, but rather as an initial gateway device that facilitates subsequent dilation. This approach may be particularly useful when advancement of conventional dilation devices is expected to be difficult because of a tight stricture or unstable endoscope positioning. This treatment strategy may contribute to more efficient endoscopic management of postoperative difficult biliary strictures in patients with surgically altered anatomy.

This study has several limitations. First, this was a retrospective study conducted at a single center. Second, the sample size was relatively small. Third, the severity of biliary strictures was not evaluated using objective criteria. Fourth, the selection of the dilation device was left to the discretion of the endoscopist, introducing the possibility of selection bias, with technically more challenging strictures being preferentially treated using the Drill Dilator. Fifth, the follow-up periods varied among patients, and some patients were transferred to other institutions shortly after dilation. Therefore, the occurrence of stricture recurrence may not have been accurately assessed in all patients. Sixth, patients in whom guidewire passage was unsuccessful were excluded, limiting the generalizability of the findings to patients in whom guidewire passage could be achieved. Finally, all procedures in the Drill Dilator group were performed using SIF-H290S, which may have influenced the procedural outcomes.

## 5. Conclusions

The Drill Dilator may serve as a feasible “initial gateway device” for facilitating the passage of subsequent devices in the treatment of postoperative biliary strictures in patients with surgically altered anatomy. Its use as an initial dilation device may facilitate subsequent endoscopic intervention. However, the present study did not demonstrate its superiority over conventional dilation devices in such patients. Larger prospective studies are needed to determine its comparative efficacy and safety.

## Figures and Tables

**Table 1:** Patient background.

	Drill Dilator	Conventional Dilator
Patients, *n*	6	12
Age, years	71.5 (65–78)	69 (42–82)
Male sex, *n*	4	7
primary disease		
Bile duct cancer	3	3
Gallbladder cancer	1	1
Pancreatic cancer	1	2
IPMC	0	2
pNET	0	1
AIH	0	1
Lymphoma	0	1
other	1	1
Type of surgery		
SSPPD	4	5
Extrahepatic bile duct resection	1	1
Extrahepatic bile duct resection. with Hepatectomy	1	1
Liver transplantation	0	2
Total pancreatectomy	0	2
Gastrojejunostomy	0	1
Intestinal reconstruction		
II-A-1	4	6
Roux-en-Y	2	4
B-II	0	1
Gastrojejunostomy	0	1

**Table 2:** Initial treatment-related characteristics and clinical outcome.

	Drill Dilator (*n* = 6)	Conventional Dilator (*n* = 12)
Stricture site		
Hepaticojejunostomy site	6	11
Distal bile duct	0	1
Etiology of stricture site		
benign	5	11
malignant	1	1
1st dilation device		
Drill	6	0
Balloon	0	8
Bougie	0	4
Scope		
SIF-H290S	6	8
PCF-PQ260L	0	2
EI-580BT	0	1
PCF-H290TI	0	1
Success rate		
Stricture access	6/6 (100%)	12/12 (100%)
Guidewire passage	6/6 (100%)	12/12 (100%)
Dilation device passage	6/6 (100%)	12/12 (100%)
Need for additional dilation	3/6 (50%)	4/12 (33.3%)
Technical success	6/6 (100%)	11/12 (91.7%)
Procedure time, min		
Total procedure time	81 (33–125)	79 (20–174)
Dilation procedure time	62 (28–98)	46 (10–127)
procedure-related Adverse event, *n* (%)	2/6 (33.3%)	2/12 (16.7%)
PEC	2	1
PEP	0	1
Severity, *n*		
Mild	2	1
Moderate	0	1
Severe	0	0
Follow-up period, day	247.5 (18–589)	920.5 (2–3344)
Recurrence of stenosis	0/6 (0%)	1/12 (8.3%)

## Data Availability

The data generated and analyzed during this study are not publicly available due to privacy and ethical restrictions.

## Supplementary Materials

The following supporting information can be downloaded at https://www.mdpi.com/article/10.3390/jcm15218666/s1, Video S1: Drill Dilator.
